# Supplementary material for: Area-Wide Elimination of Subterranean Termite Colonies Using a Novaluron Bait
Source: Insects. 2021 Feb 24;12(3):192. doi: 10.3390/insects12030192 (PMC7996135; doi:10.3390/insects12030192)
Supplement: Supplementary file 1 [file insects-12-00192-s001.zip › Supp_files/Fig_S1.pdf]

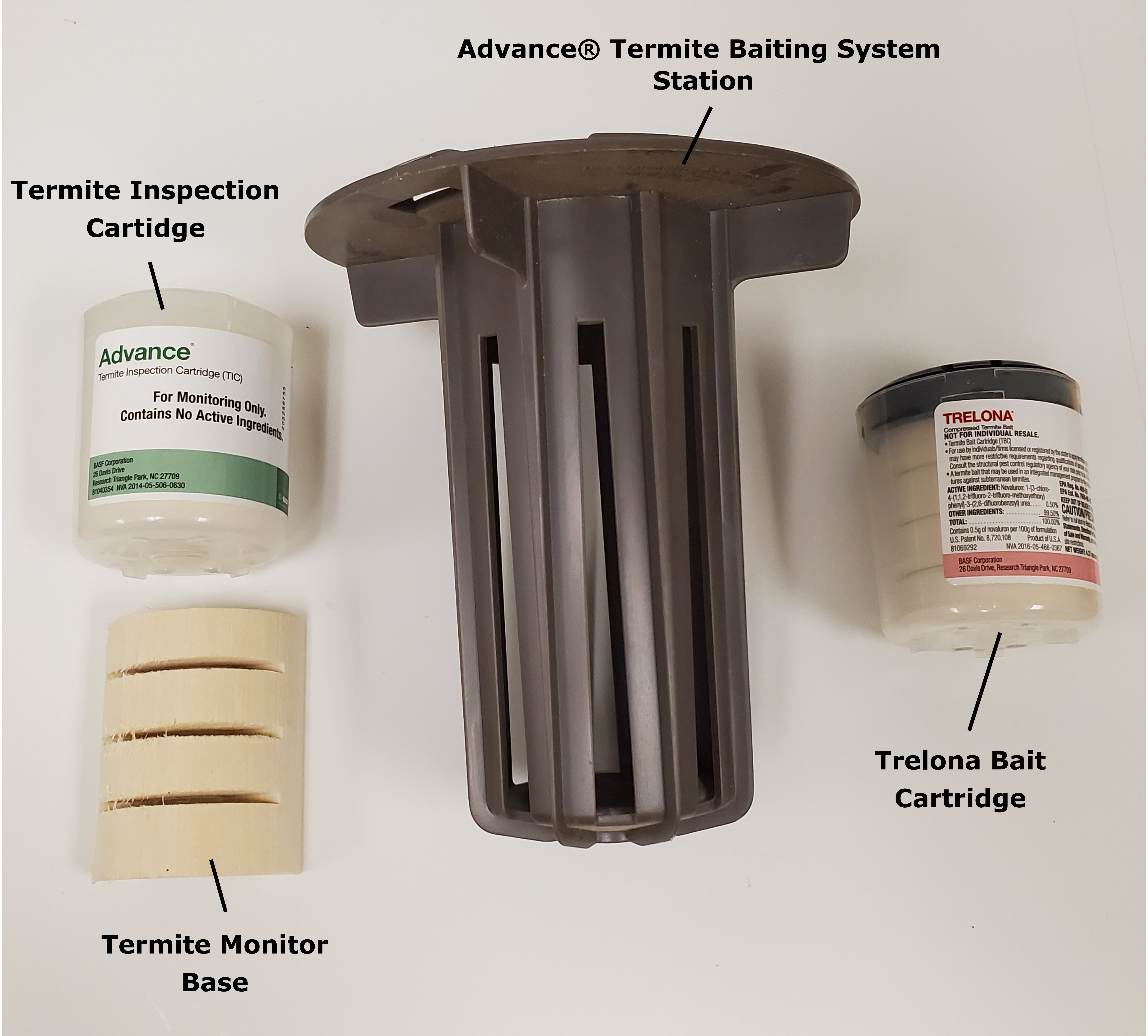

Figure S1: The ATBS® monitoring stations with the various inserts. All pre-treatment stations contained a termite inspection cartridge and a termite monitor base (left). In May 2018, stations within the wooded area of the treatment site had these inserts replaced with two Trelona® bait cartridges (right). The termite bait was left in these stations for the remainder of the study. The rest of the stations in the treatment site as well as all of the stations in the control site continued with each station having an inspection cartridge and a wooden monitor base.
