# Supplementary material for: Area-Wide Elimination of Subterranean Termite Colonies Using a Novaluron Bait
Source: Insects. 2021 Feb 24;12(3):192. doi: 10.3390/insects12030192 (PMC7996135; doi:10.3390/insects12030192)
Supplement: Supplementary file 1 [file insects-12-00192-s001.zip › Supp_files/tableS1.pdf]

Control Site

Colony.year	1.1	1.2	1.3	1.4	2.1	2.3	2.4	3.1	3.3	3.4	4.1	5.1	5.3	5.4	6.1	6.2	6.3	6.4	7.2	8.2	8.3	8.4	9.2	10.2	10.3	10.4	11.3	11.4	12.3	13.3	13.4	14.3	14.4	15.4	16.4	17.4	v.1	v.2	v.3	v.4
1.1		0.93	0.98	0.92	0.00	0.00	0.00	0.00	0.00	0.00	0.00	0.00	0.00	0.00	0.00	0.00	0.00	0.00	0.00	0.00	0.00	0.00	0.00	0.00	0.00	0.00	0.00	0.00	0.00	0.00	0.00	0.00	0.00	0.00	0.00	0.00	0.0	0.0	0.0	0.0
1.2	-0.0492		0.75	0.94	0.00	0.00	0.00	0.00	0.00	0.00	0.00	0.00	0.00	0.00	0.00	0.00	0.00	0.00	0.00	0.00	0.00	0.00	0.00	0.00	0.00	0.00	0.00	0.00	0.00	0.00	0.00	0.00	0.00	0.00	0.00	0.00	0.0	0.0	0.0	0.0
1.3	-0.0739	-0.0273		0.83	0.00	0.00	0.00	0.00	0.00	0.00	0.00	0.00	0.00	0.00	0.00	0.00	0.00	0.00	0.00	0.00	0.00	0.00	0.00	0.00	0.00	0.00	0.00	0.00	0.00	0.00	0.00	0.00	0.00	0.00	0.00	0.00	0.0	0.0	0.0	0.0
1.4	-0.0443	-0.0262	-0.0200		0.00	0.00	0.00	0.00	0.00	0.00	0.00	0.00	0.00	0.00	0.00	0.00	0.00	0.00	0.00	0.00	0.00	0.00	0.00	0.00	0.00	0.00	0.00	0.00	0.00	0.00	0.00	0.00	0.00	0.00	0.00	0.00	0.0	0.0	0.0	0.0
2.1	0.3107	0.3184	0.3125	0.3402		0.20	0.69	0.00	0.00	0.00	0.00	0.00	0.00	0.00	0.00	0.00	0.00	0.00	0.00	0.00	0.00	0.00	0.00	0.00	0.00	0.00	0.00	0.00	0.00	0.00	0.00	0.00	0.00	0.00	0.00	0.00	0.0	0.0	0.0	0.0
2.3	0.2991	0.2999	0.2988	0.3198	0.0028		0.70	0.00	0.00	0.00	0.00	0.00	0.00	0.00	0.00	0.00	0.00	0.00	0.00	0.00	0.00	0.00	0.00	0.00	0.00	0.00	0.00	0.00	0.00	0.00	0.00	0.00	0.00	0.00	0.00	0.00	0.0	0.0	0.0	0.0
2.4	0.3045	0.3103	0.3058	0.3323	-0.0052	-0.0037		0.00	0.00	0.00	0.00	0.00	0.00	0.00	0.00	0.00	0.00	0.00	0.00	0.00	0.00	0.00	0.00	0.00	0.00	0.00	0.00	0.00	0.00	0.00	0.00	0.00	0.00	0.00	0.00	0.00	0.0	0.0	0.0	0.0
3.1	0.4259	0.4158	0.4249	0.4400	0.3200	0.2933	0.3092		0.92	0.78	0.00	0.00	0.00	0.00	0.00	0.00	0.00	0.00	0.00	0.00	0.00	0.00	0.00	0.00	0.00	0.00	0.00	0.00	0.00	0.00	0.00	0.00	0.00	0.00	0.00	0.00	0.0	0.0	0.0	0.0
3.3	0.4292	0.4177	0.4282	0.4431	0.3195	0.2920	0.3084	-0.0080		0.89	0.00	0.00	0.00	0.00	0.00	0.00	0.00	0.00	0.00	0.00	0.00	0.00	0.00	0.00	0.00	0.00	0.00	0.00	0.00	0.00	0.00	0.00	0.00	0.00	0.00	0.00	0.0	0.0	0.0	0.0
3.4	0.3986	0.3951	0.4032	0.4252	0.2847	0.2672	0.2760	-0.0127	-0.0142		0.00	0.00	0.00	0.00	0.00	0.00	0.00	0.00	0.00	0.00	0.00	0.00	0.00	0.00	0.00	0.00	0.00	0.00	0.00	0.00	0.00	0.00	0.00	0.00	0.00	0.00	0.0	0.0	0.0	0.0
4.1	0.4288	0.4549	0.4362	0.4638	0.3544	0.3446	0.3498	0.4832	0.4899	0.4624		0.00	0.00	0.00	0.00	0.00	0.00	0.00	0.00	0.00	0.00	0.00	0.00	0.00	0.00	0.00	0.00	0.00	0.00	0.00	0.00	0.00	0.00	0.00	0.00	0.00	0.0	0.0	0.0	0.0
5.1	0.4619	0.4769	0.4642	0.4829	0.3844	0.3682	0.3797	0.4957	0.5015	0.4827	0.2776		0.98	0.21	0.00	0.00	0.00	0.00	0.00	0.00	0.00	0.00	0.00	0.00	0.00	0.00	0.00	0.00	0.00	0.00	0.00	0.00	0.00	0.00	0.00	0.00	0.0	0.0	0.0	0.0
5.3	0.4598	0.4761	0.4628	0.4826	0.3814	0.3656	0.3766	0.4959	0.5018	0.4823	0.2780	-0.0159		0.49	0.00	0.00	0.00	0.00	0.00	0.00	0.00	0.00	0.00	0.00	0.00	0.00	0.00	0.00	0.00	0.00	0.00	0.00	0.00	0.00	0.00	0.00	0.0	0.0	0.0	0.0
5.4	0.4882	0.4969	0.4885	0.5007	0.4175	0.3959	0.4133	0.5079	0.5127	0.5020	0.3172	-0.0006	-0.0058		0.00	0.00	0.00	0.00	0.00	0.00	0.00	0.00	0.00	0.00	0.00	0.00	0.00	0.00	0.00	0.00	0.00	0.00	0.00	0.00	0.00	0.00	0.0	0.0	0.0	0.0
6.1	0.4795	0.5037	0.4890	0.5139	0.3977	0.3839	0.3924	0.4773	0.4861	0.4580	0.3409	0.4716	0.4720	0.5008		0.95	0.95	1.00	0.00	0.00	0.00	0.00	0.00	0.00	0.00	0.00	0.00	0.00	0.00	0.00	0.00	0.00	0.00	0.00	0.00	0.08	0.0	0.0	0.0	0.0
6.2	0.5369	0.5428	0.5375	0.5469	0.4535	0.4236	0.4484	0.4942	0.5018	0.4833	0.3910	0.5113	0.5131	0.5266	-0.0575		0.77	0.70	0.00	0.00	0.00	0.00	0.00	0.00	0.00	0.00	0.00	0.00	0.00	0.00	0.00	0.00	0.00	0.00	0.00	0.00	0.0	0.0	0.0	0.0
6.3	0.5295	0.5360	0.5295	0.5383	0.4646	0.4380	0.4622	0.4847	0.4902	0.4704	0.3869	0.5061	0.5076	0.5230	-0.0498	-0.0129		0.86	0.00	0.00	0.00	0.00	0.00	0.00	0.00	0.00	0.00	0.00	0.00	0.00	0.00	0.00	0.00	0.00	0.00	0.00	0.0	0.0	0.0	0.0
6.4	0.5181	0.5260	0.5184	0.5289	0.4477	0.4214	0.4445	0.4806	0.4865	0.4649	0.3674	0.4918	0.4933	0.5112	-0.0578	-0.0139	-0.0098		0.00	0.00	0.00	0.00	0.00	0.00	0.00	0.00	0.00	0.00	0.00	0.00	0.00	0.00	0.00	0.00	0.00	0.00	0.0	0.0	0.0	0.0
7.2	0.4230	0.4502	0.4313	0.4599	0.3444	0.3346	0.3402	0.4778	0.4842	0.4583	0.1344	0.2689	0.2724	0.3145	0.4163	0.4731	0.4616	0.4439		0.01	0.00	0.00	0.00	0.00	0.00	0.00	0.00	0.00	0.00	0.00	0.00	0.00	0.00	0.00	0.00	0.00	0.0	0.0	0.0	0.0
8.2	0.4839	0.4975	0.4863	0.5040	0.3678	0.3592	0.3664	0.5140	0.5202	0.5054	0.3361	0.4259	0.4292	0.4581	0.4854	0.5199	0.5073	0.4956	0.3318		0.87	0.28	0.00	0.00	0.00	0.00	0.00	0.00	0.00	0.00	0.00	0.00	0.00	0.00	0.00	0.00	0.0	0.0	0.0	0.0
8.3	0.4800	0.4904	0.4807	0.4956	0.3707	0.3613	0.3688	0.5032	0.5083	0.4951	0.3241	0.4131	0.4175	0.4440	0.4774	0.5040	0.4963	0.4841	0.3192	-0.0121		0.09	0.00	0.00	0.00	0.00	0.00	0.00	0.00	0.00	0.00	0.00	0.00	0.00	0.00	0.00	0.0	0.0	0.0	0.0
8.4	0.4843	0.4934	0.4846	0.4978	0.3885	0.3758	0.3857	0.5056	0.5105	0.5001	0.3299	0.4031	0.4095	0.4366	0.4749	0.4982	0.4885	0.4768	0.3072	0.0131	0.0093		0.00	0.00	0.00	0.00	0.00	0.00	0.00	0.00	0.00	0.00	0.00	0.00	0.00	0.00	0.0	0.0	0.0	0.0
9.2	0.3400	0.3850	0.3493	0.3844	0.3464	0.3393	0.3423	0.4753	0.4816	0.4458	0.1942	0.2275	0.2284	0.2597	0.3363	0.4280	0.4193	0.3983	0.1882	0.3718	0.3622	0.3493		0.00	0.00	0.00	0.00	0.00	0.00	0.00	0.00	0.00	0.00	0.00	0.00	0.00	0.00	0.00	0.00	0.00
10.2	0.4694	0.4879	0.4750	0.4960	0.3865	0.3719	0.3819	0.4825	0.4904	0.4667	0.3541	0.3451	0.3533	0.4086	0.3370	0.4066	0.3755	0.3642	0.2734	0.3018	0.2894	0.2409	0.3013		0.71	0.90	0.00	0.00	0.00	0.00	0.00	0.00	0.00	0.00	0.00	0.00	0.00	0.00	0.00	0.00
10.3	0.5334	0.5382	0.5330	0.5378	0.4626	0.4372	0.4665	0.5158	0.5200	0.5097	0.4209	0.4168	0.4222	0.4693	0.3877	0.4425	0.4078	0.4038	0.3220	0.3503	0.3343	0.2799	0.3937	-0.0134		0.49	0.00	0.00	0.00	0.00	0.00	0.00	0.00	0.00	0.00	0.00	0.00	0.00	0.00	0.00
10.4	0.4996	0.5092	0.5013	0.5141	0.4141	0.3923	0.4104	0.4935	0.5006	0.4843	0.3809	0.3538	0.3621	0.4148	0.3661	0.4232	0.3915	0.3826	0.2948	0.3593	0.3420	0.2965	0.3453	-0.0159	0.0009		0.00	0.00	0.00	0.00	0.00	0.00	0.00	0.00	0.00	0.00	0.00	0.00	0.00	0.00
11.3	0.3279	0.3515	0.3293	0.3503	0.3170	0.3130	0.3143	0.3197	0.3271	0.2881	0.2850	0.3537	0.3556	0.3928	0.1548	0.1827	0.1792	0.1724	0.2961	0.3500	0.3500	0.3435	0.2376	0.2046	0.2586	0.2231		0.41	0.00	0.00	0.00	0.00	0.00	0.00	0.00	0.00	0.00	0.00	0.00	0.00
11.4	0.3236	0.3538	0.3266	0.3527	0.3148	0.3108	0.3120	0.3168	0.3269	0.2798	0.2995	0.3652	0.3650	0.4059	0.1775	0.2166	0.2174	0.2087	0.3118	0.3700	0.3730	0.3730	0.2419	0.2493	0.3219	0.2745	0.0023		0.00	0.00	0.00	0.00	0.00	0.00	0.00	0.00	0.00	0.00	0.00	0.00
12.3	0.4180	0.4476	0.4263	0.4573	0.3192	0.3127	0.3176	0.4755	0.4814	0.4522	0.3913	0.4267	0.4240	0.4556	0.4404	0.4954	0.4929	0.4792	0.3834	0.4551	0.4581	0.4658	0.3772	0.4226	0.4704	0.4478	0.3460	0.3438		0.00	0.00	0.00	0.00	0.00	0.00	0.00	0.00	0.00	0.00	0.00
13.3	0.5000	0.5135	0.5055	0.5219	0.4101	0.3917	0.4041	0.5319	0.5395	0.5317	0.2954	0.3730	0.3702	0.4058	0.4531	0.4786	0.4696	0.4593	0.4061	0.4636	0.4577	0.4828	0.4526	0.4601	0.5046	0.4596	0.3683	0.3774	0.4618		0.81	0.00	0.00	0.00	0.00	0.00	0.00	0.00	0.00	0.00
13.4	0.5120	0.5182	0.5120	0.5221	0.4374	0.4108	0.4323	0.5264	0.5316	0.5256	0.3004	0.3924	0.3892	0.4186	0.4495	0.4599	0.4583	0.4495	0.4237	0.4683	0.4624	0.4842	0.4781	0.4671	0.5027	0.4620	0.3853	0.4024	0.4809	-0.0181		0.00	0.00	0.00	0.00	0.00	0.00	0.00	0.00	0.00
14.3	0.4884	0.5033	0.4910	0.5091	0.4101	0.3906	0.4053	0.4584	0.4718	0.4565	0.4302	0.4638	0.4659	0.4943	0.4846	0.5278	0.5159	0.5055	0.4034	0.4646	0.4545	0.4465	0.4227	0.4016	0.4502	0.4240	0.2727	0.2629	0.3809	0.5164	0.5208		0.99	0.00	0.00	0.00	0.00	0.00	0.00	0.00
14.4	0.5100	0.5188	0.5103	0.5215	0.4470	0.4239	0.4444	0.4630	0.4750	0.4675	0.4592	0.4854	0.4875	0.5074	0.5027	0.5307	0.5208	0.5136	0.4338	0.4833	0.4735	0.4641	0.4563	0.4293	0.4620	0.4441	0.3038	0.2943	0.4075	0.5275	0.5272	-0.0226		0.00	0.00	0.00	0.00	0.00	0.00	0.00
15.4	0.4445	0.4607	0.4463	0.4654	0.3121	0.3189	0.3179	0.4804	0.4851	0.4625	0.4036	0.2590	0.2611	0.2827	0.4252	0.4796	0.4814	0.4673	0.3929	0.4138	0.4083	0.4185	0.3376	0.3802	0.4622	0.3997	0.3491	0.3538	0.4035	0.3932	0.4157	0.4642	0.4892		0.00	0.00	0.00	0.00	0.00	0.00
16.4	0.4236	0.4539	0.4318	0.4629	0.3277	0.3228	0.3262	0.4839	0.4905	0.4580	0.2887	0.3635	0.3601	0.3916	0.4429	0.5090	0.5062	0.4908	0.2933	0.4253	0.4201	0.4264	0.2834	0.4351	0.5099	0.4693	0.3482	0.3473	0.3523	0.4653	0.4862	0.4578	0.4861	0.4158		0.00	0.00	0.00	0.00	0.00
17.4	0.3090	0.3463	0.3118	0.3343	0.3584	0.3486	0.3579	0.4276	0.4320	0.4015	0.3904	0.3192	0.3195	0.3422	0.3465	0.4024	0.4053	0.3929	0.3764	0.4348	0.4365	0.4380	0.2119	0.3534	0.4292	0.3758	0.2117	0.2051	0.3782	0.4175	0.4370	0.4427	0.4708	0.3048	0.3959		0.00	0.00	0.00	0.00
v.1	0.3652	0.3795	0.3658	0.3835	0.2770	0.2844	0.2779	0.3509	0.3534	0.3396	0.3078	0.3610	0.3601	0.3856	0.3809	0.4122	0.4264	0.4104	0.3186	0.3268	0.3298	0.3538	0.3331	0.3645	0.4245	0.3838	0.3095	0.3059	0.3377	0.3333	0.3502	0.3826	0.4132	0.3117	0.3395	0.3427		0.82	0.74	0.93
v.2	0.3640	0.3752	0.3641	0.3788	0.2724	0.2811	0.2735	0.3427	0.3452	0.3376	0.3087	0.3578	0.3574	0.3768	0.3797	0.4024	0.4108	0.3984	0.3193	0.3206	0.3205	0.3423	0.3332	0.3627	0.4092	0.3786	0.3097	0.3065	0.3378	0.3331	0.3425	0.3779	0.3999	0.3019	0.3393	0.3399	-0.0017		0.24	0.49
v.3	0.3702	0.3802	0.3702	0.3838	0.2828	0.2900	0.2828	0.3463	0.3490	0.3440	0.3137	0.3632	0.3633	0.3792	0.3865	0.4047	0.4095	0.3993	0.3263	0.3269	0.3257	0.3460	0.3400	0.3688	0.4080	0.3833	0.3164	0.3134	0.3462	0.3358	0.3417	0.3827	0.3996	0.3110	0.3461	0.3453	-0.0012	0.0006		0.99
v.4	0.3706	0.3839	0.3711	0.3879	0.2826	0.2894	0.2831	0.3559	0.3585	0.3463	0.3136	0.3658	0.3651	0.3883	0.3868	0.4148	0.4273	0.4123	0.3253	0.3314	0.3333	0.3564	0.3391	0.3698	0.4254	0.3879	0.3148	0.3117	0.3447	0.3384	0.3529	0.3869	0.4147	0.3158	0.3454	0.3474	-0.0026	-0.0003	-0.0021	

Table S1: Pairwise matrix of genetic differentiation for each colony by year in the control site. F_{ST} values between each pair of trials are provided below the diagonal, values of the pairwise G-tests of genetic differentiation are indicated above the diagonal.
