# Supplementary material for: Area-Wide Elimination of Subterranean Termite Colonies Using a Novaluron Bait
Source: Insects. 2021 Feb 24;12(3):192. doi: 10.3390/insects12030192 (PMC7996135; doi:10.3390/insects12030192)
Supplement: Supplementary file 1 [file insects-12-00192-s001.zip › Supp_files/tableS2.pdf]

Treatment Site

Colony.year	1.1	2.1	2.2	2.3	2.4	3.1	3.2	3.3	4.1	5.1	5.2	5.3	6.1	6.2	6.3	7.1	7.2	7.3	8.1	8.2	8.3	9.2	9.3	10.2	10.3	11.2	11.3	11.4	12.3	13.3	13.4	14.4	15.4	v.1	v.2	v.3
1.1		0.00	0.00	0.00	0.00	0.00	0.00	0.00	0.00	0.00	0.00	0.00	0.00	0.00	0.00	0.00	0.00	0.00	0.00	0.00	0.00	0.00	0.00	0.00	0.00	0.00	0.00	0.00	0.00	0.00	0.00	0.00	0.00	0.00	0.00	0.00
2.1	0.5899		0.53	0.09	0.82	0.00	0.00	0.00	0.00	0.00	0.00	0.00	0.00	0.00	0.00	0.00	0.00	0.00	0.00	0.00	0.00	0.00	0.00	0.00	0.00	0.00	0.00	0.00	0.00	0.00	0.00	0.00	0.00	0.00	0.00	0.00
2.2	0.5753	0.0044		0.15	1.00	0.00	0.00	0.00	0.00	0.00	0.00	0.00	0.00	0.00	0.00	0.00	0.00	0.00	0.00	0.00	0.00	0.00	0.00	0.00	0.00	0.00	0.00	0.00	0.00	0.00	0.00	0.00	0.00	0.00	0.00	0.00
2.3	0.5840	-0.0032	0.0042		0.45	0.00	0.00	0.00	0.00	0.00	0.00	0.00	0.00	0.00	0.00	0.00	0.00	0.00	0.00	0.00	0.00	0.01	0.00	0.00	0.00	0.00	0.00	0.00	0.00	0.00	0.00	0.00	0.01	0.00	0.00	0.00
2.4	0.5847	-0.0120	-0.0242	-0.0136		0.00	0.00	0.00	0.00	0.00	0.00	0.00	0.00	0.00	0.00	0.00	0.00	0.00	0.00	0.00	0.00	0.00	0.00	0.00	0.00	0.00	0.00	0.00	0.00	0.00	0.00	0.00	0.01	0.00	0.00	0.00
3.1	0.4954	0.3915	0.3341	0.3554	0.3369		0.88	0.80	0.00	0.00	0.00	0.00	0.09	0.00	0.00	0.00	0.00	0.05	0.00	0.00	0.00	0.00	0.00	0.00	0.00	0.00	0.00	0.00	0.00	0.00	0.00	0.00	0.00	0.00	0.00	0.00
3.2	0.4796	0.3641	0.2972	0.3184	0.2955	-0.0130		1.00	0.02	0.00	0.00	0.00	0.00	0.00	0.00	0.00	0.00	0.00	0.00	0.00	0.00	0.01	0.01	0.00	0.00	0.00	0.00	0.00	0.00	0.00	0.00	0.00	0.00	0.00	0.00	0.00
3.3	0.4714	0.3589	0.2986	0.3235	0.3003	-0.0049	-0.0227		0.00	0.00	0.00	0.00	0.06	0.00	0.00	0.00	0.00	0.02	0.00	0.00	0.00	0.00	0.00	0.00	0.00	0.00	0.00	0.00	0.00	0.00	0.00	0.00	0.00	0.00	0.00	0.00
4.1	0.5611	0.2872	0.2291	0.2818	0.2410	0.3156	0.2689	0.2704		0.00	0.00	0.00	0.01	0.00	0.00	0.00	0.00	0.00	0.04	0.02	0.01	0.00	0.00	0.06	0.10	0.00	0.00	0.00	0.00	0.00	0.00	0.00	0.01	0.00	0.00	0.00
5.1	0.4990	0.4539	0.4036	0.3893	0.3903	0.3697	0.3351	0.3545	0.4286		0.70	0.14	0.00	0.00	0.00	0.00	0.00	0.00	0.00	0.00	0.00	0.00	0.00	0.00	0.00	0.02	0.00	0.00	0.00	0.00	0.00	0.00	0.00	0.00	0.00	0.00
5.2	0.4301	0.4256	0.3831	0.3752	0.3776	0.3572	0.3358	0.3455	0.3958	-0.0112		0.14	0.00	0.00	0.00	0.00	0.00	0.00	0.00	0.00	0.00	0.00	0.00	0.00	0.00	0.00	0.00	0.00	0.00	0.00	0.00	0.00	0.00	0.00	0.00	0.00
5.3	0.4015	0.4417	0.4015	0.3936	0.3998	0.3716	0.3569	0.3596	0.4102	0.0168	-0.0002		0.00	0.00	0.00	0.00	0.00	0.00	0.00	0.00	0.00	0.00	0.00	0.00	0.00	0.00	0.00	0.00	0.00	0.00	0.00	0.00	0.00	0.00	0.00	0.00
6.1	0.5515	0.3535	0.2791	0.3188	0.2818	0.1839	0.1459	0.1613	0.0638	0.3355	0.3322	0.3431		0.13	0.98	0.00	0.00	0.00	0.00	0.00	0.00	0.00	0.00	0.01	0.02	0.00	0.00	0.00	0.00	0.00	0.00	0.00	0.00	0.00	0.00	0.00
6.2	0.5444	0.3614	0.2908	0.3325	0.2949	0.2279	0.1891	0.2037	0.0804	0.3502	0.3376	0.3402	-0.0065		0.01	0.00	0.00	0.00	0.00	0.00	0.00	0.00	0.00	0.00	0.00	0.00	0.01	0.00	0.00	0.00	0.00	0.00	0.00	0.00	0.00	0.00
6.3	0.5100	0.3539	0.2917	0.3315	0.2949	0.1960	0.1638	0.1801	0.1032	0.3428	0.3293	0.3205	-0.0334	0.0117		0.00	0.00	0.03	0.00	0.00	0.00	0.00	0.00	0.00	0.00	0.00	0.00	0.00	0.00	0.00	0.00	0.00	0.00	0.00	0.00	0.00
7.1	0.5409	0.3097	0.2426	0.2713	0.2418	0.0824	0.0551	0.0626	0.1736	0.3180	0.3202	0.3383	0.1158	0.1615	0.1390		0.52	0.34	0.02	0.02	0.00	0.00	0.00	0.00	0.00	0.00	0.00	0.00	0.00	0.00	0.00	0.00	0.00	0.00	0.00	0.00
7.2	0.5332	0.3005	0.2444	0.2591	0.2392	0.1552	0.1239	0.1341	0.2248	0.3046	0.3106	0.3287	0.1423	0.2090	0.1622	0.0197		0.71	0.00	0.00	0.00	0.00	0.00	0.00	0.00	0.00	0.00	0.00	0.00	0.00	0.00	0.00	0.01	0.00	0.00	0.00
7.3	0.5457	0.3010	0.2351	0.2660	0.2349	0.1467	0.1130	0.1224	0.1595	0.3252	0.3261	0.3441	0.0902	0.1571	0.1143	0.0231	-0.0086		0.00	0.00	0.00	0.00	0.00	0.00	0.00	0.00	0.00	0.00	0.00	0.00	0.00	0.00	0.01	0.00	0.00	0.00
8.1	0.6080	0.3968	0.3289	0.3713	0.3391	0.2933	0.2630	0.2659	0.2227	0.4335	0.4098	0.4262	0.2058	0.2180	0.2263	0.1940	0.2553	0.2146		0.47	0.69	0.02	0.00	0.00	0.00	0.00	0.00	0.00	0.01	0.00	0.00	0.00	0.00	0.00	0.00	0.00
8.2	0.5893	0.3310	0.2599	0.3064	0.2672	0.2712	0.2277	0.2361	0.1324	0.4100	0.3919	0.4074	0.1620	0.1804	0.1967	0.1316	0.1894	0.1530	0.0040		0.62	0.01	0.00	0.00	0.00	0.00	0.00	0.00	0.02	0.00	0.00	0.00	0.00	0.00	0.00	0.00
8.3	0.5753	0.3630	0.2985	0.3395	0.3060	0.2865	0.2518	0.2583	0.2033	0.4155	0.3944	0.4040	0.1997	0.2140	0.2258	0.1673	0.2227	0.1934	-0.0149	-0.0057		0.00	0.00	0.00	0.00	0.00	0.00	0.00	0.00	0.00	0.00	0.00	0.00	0.00	0.00	0.00
9.2	0.4065	0.2946	0.2169	0.2225	0.2157	0.2402	0.1868	0.2079	0.2863	0.2948	0.3033	0.3233	0.2252	0.2419	0.2476	0.2041	0.2167	0.2075	0.1870	0.1803	0.1796		0.64	0.00	0.00	0.00	0.00	0.00	0.01	0.00	0.00	0.00	0.00	0.00	0.00	0.00
9.3	0.4667	0.3288	0.2428	0.2638	0.2464	0.2432	0.2049	0.2196	0.2882	0.3292	0.3266	0.3421	0.2192	0.2312	0.2384	0.2178	0.2497	0.2239	0.1715	0.1757	0.1727	0.0044		0.00	0.00	0.00	0.00	0.00	0.00	0.00	0.00	0.00	0.00	0.00	0.00	0.00
10.2	0.6313	0.5188	0.4714	0.4764	0.4951	0.3583	0.3641	0.3465	0.4694	0.4944	0.4455	0.4687	0.3429	0.3322	0.3138	0.3747	0.4148	0.3832	0.3366	0.3080	0.3098	0.3181	0.2422		1.00	0.00	0.00	0.00	0.00	0.00	0.00	0.00	0.00	0.00	0.00	0.00
10.3	0.6244	0.5081	0.4556	0.4607	0.4757	0.3376	0.3369	0.3257	0.4461	0.4721	0.4321	0.4559	0.3137	0.3066	0.2926	0.3521	0.3928	0.3607	0.3183	0.2902	0.2950	0.2983	0.2275	-0.0320		0.00	0.00	0.00	0.00	0.00	0.00	0.00	0.00	0.00	0.00	0.00
11.2	0.4771	0.3016	0.2300	0.2515	0.2200	0.2364	0.1773	0.2027	0.1158	0.2361	0.2518	0.2701	0.0826	0.0693	0.1064	0.1249	0.1493	0.1223	0.2036	0.1516	0.1921	0.1725	0.1946	0.3643	0.3363		0.97	1.00	0.00	0.00	0.00	0.00	0.01	0.00	0.00	0.00
11.3	0.4859	0.2934	0.2245	0.2618	0.2233	0.2323	0.1830	0.2020	0.0903	0.2879	0.2825	0.2866	0.0789	0.0674	0.1079	0.1296	0.1672	0.1275	0.1878	0.1434	0.1827	0.1912	0.1979	0.3164	0.2974	-0.0429		0.94	0.00	0.00	0.00	0.00	0.00	0.00	0.00	0.00
11.4	0.5068	0.3279	0.2511	0.2833	0.2462	0.2312	0.1812	0.2021	0.1088	0.2643	0.2704	0.2844	0.0741	0.0622	0.0978	0.1336	0.1734	0.1342	0.1925	0.1525	0.1878	0.1859	0.1895	0.3329	0.3082	-0.0642	-0.0279		0.00	0.00	0.00	0.00	0.00	0.00	0.00	0.00
12.3	0.5396	0.3214	0.2664	0.2696	0.2593	0.1827	0.1499	0.1661	0.2775	0.3116	0.3184	0.3385	0.1433	0.2165	0.1618	0.1474	0.0917	0.1026	0.2620	0.2193	0.2414	0.1939	0.2286	0.3608	0.3350	0.1781	0.2015	0.2052		0.00	0.00	0.01	0.01	0.00	0.00	0.00
13.3	0.5737	0.3487	0.3377	0.2984	0.3223	0.4211	0.4072	0.4090	0.4807	0.3963	0.3782	0.3927	0.4378	0.4428	0.4299	0.3864	0.3539	0.3648	0.4844	0.4641	0.4621	0.3726	0.3929	0.5293	0.5144	0.3517	0.3684	0.3749	0.3884		1.00	0.00	0.00	0.00	0.00	0.00
13.4	0.5753	0.3498	0.3317	0.2911	0.3139	0.4070	0.3859	0.3928	0.4840	0.3683	0.3626	0.3842	0.4195	0.4271	0.4154	0.3651	0.3322	0.3464	0.4744	0.4512	0.4508	0.3464	0.3720	0.5378	0.5187	0.3188	0.3443	0.3451	0.3631	-0.0350		0.00	0.00	0.00	0.00	0.00
14.4	0.4840	0.3437	0.2859	0.2966	0.2791	0.1940	0.1651	0.1790	0.1985	0.2723	0.2748	0.2757	0.0869	0.1116	0.0852	0.1120	0.1002	0.1068	0.3063	0.2684	0.2924	0.2333	0.2572	0.3996	0.3803	0.1083	0.1285	0.1267	0.1541	0.3861	0.3628		0.00	0.00	0.00	0.00
15.4	0.6004	0.3243	0.2923	0.2882	0.2839	0.3783	0.3319	0.3442	0.3635	0.3696	0.3714	0.3957	0.3459	0.3647	0.3616	0.2831	0.2529	0.2775	0.4170	0.3483	0.3814	0.3016	0.3600	0.5479	0.5279	0.2505	0.2824	0.2978	0.2659	0.4460	0.4249	0.2862		0.00	0.00	0.00
v.1	0.5520	0.4577	0.4095	0.3957	0.3998	0.3126	0.2890	0.3024	0.4675	0.3237	0.3297	0.3515	0.3784	0.3916	0.3850	0.2917	0.3010	0.3273	0.4096	0.3977	0.3925	0.2842	0.3196	0.5028	0.4816	0.2996	0.3303	0.3243	0.3113	0.4022	0.3785	0.3048	0.3809		0.84	0.89
v.2	0.4794	0.4094	0.3767	0.3725	0.3732	0.3077	0.2926	0.3020	0.4101	0.3282	0.3196	0.3228	0.3629	0.3711	0.3705	0.2951	0.3046	0.3233	0.3625	0.3626	0.3532	0.2790	0.3097	0.4288	0.4189	0.3077	0.3300	0.3283	0.3084	0.3754	0.3609	0.3090	0.3698	-0.0171		0.24
v.3	0.5095	0.4245	0.3849	0.3784	0.3792	0.3286	0.3092	0.3203	0.4212	0.3297	0.3235	0.3309	0.3675	0.3778	0.3783	0.3084	0.3114	0.3298	0.3772	0.3739	0.3680	0.2832	0.3140	0.4427	0.4303	0.3090	0.3323	0.3297	0.3114	0.3831	0.3655	0.3160	0.3735	-0.0143	0.0028	

Table S2: Pairwise matrix of genetic differentiation for each colony by year in the treatment site. F_{ST} values between each pair of trials are provided below the diagonal, values of the pairwise G-tests of genetic differentiation are indicated above the diagonal.
